# Treatment Outcomes in Adult Tuberculous Meningitis: A Systematic Review and Meta-analysis

**DOI:** 10.1093/ofid/ofaa257

**Published:** 2020-06-30

**Authors:** Anna M Stadelman, Jayne Ellis, Thomas H A Samuels, Ernest Mutengesa, Joanna Dobbin, Kenneth Ssebambulidde, Morris K Rutakingirwa, Lillian Tugume, David R Boulware, Daniel Grint, Fiona V Cresswell

**Affiliations:** 1 Division of Epidemiology and Community Health, School of Public Health, University of Minnesota, Minneapolis, Minnesota, USA; 2 Hospital for Tropical Diseases, University College London Hospitals NHS Foundation Trust, London, UK; 3 University College London Hospitals NHS Foundation Trust, London, UK; 4 Hillingdon Hospital, The Hillingdon Hospitals NHS Foundation Trust, Uxbridge, UK; 5 Clinical Research Department, London School of Hygiene and Tropical Medicine, London, UK; 6 Infectious Diseases Institute, Makerere University, Kampala, Uganda; 7 MRC-UVRI-London School of Hygiene and Tropical Medicine Uganda Research Unit, Entebbe, Uganda; 8 Division of Infectious Diseases and International Medicine, Department of Medicine, University of Minnesota, Minneapolis, Minnesota, USA; 9 Tropical Epidemiology Group, Department of Infectious Disease Epidemiology, London School of Hygiene and Tropical Medicine, London, UK

**Keywords:** tuberculous meningitis, mortality, neurological sequelae, systematic review, meta-analysis

## Abstract

**Background:**

There is substantial variation in the reported treatment outcomes for adult tuberculous meningitis (TBM). Data on survival and neurological disability by continent and HIV serostatus are scarce.

**Methods:**

We performed a systematic review and meta-analysis to characterize treatment outcomes for adult TBM. Following a systematic literature search (MEDLINE and EMBASE), studies underwent duplicate screening by independent reviewers in 2 stages to assess eligibility for inclusion. Two independent reviewers extracted data from included studies. We employed a random effects model for all meta-analyses. We evaluated heterogeneity by the *I*^2^ statistic.

**Results:**

We assessed 2197 records for eligibility; 39 primary research articles met our inclusion criteria, reporting on treatment outcomes for 5752 adults with TBM. The commonest reported outcome measure was 6-month mortality. Pooled 6-month mortality was 24% and showed significant heterogeneity (*I*^2^ > 95%; *P* < .01). Mortality ranged from 2% to 67% in Asian studies and from 23% to 80% in Sub-Saharan African studies. Mortality was significantly worse in HIV-positive adults at 57% (95% CI, 48%–67%), compared with 16% (95% CI, 10%–24%) in HIV-negative adults (*P* < .01). Physical disability was reported in 32% (95% CI, 22%–43%) of adult TBM survivors. There was considerable heterogeneity between studies in all meta-analyses, with *I*^2^ statistics consistently >50%.

**Conclusions:**

Mortality in adult TBM is high and varies considerably by continent and HIV status. The highest mortality is among HIV-positive adults in Sub-Saharan Africa. Standardized reporting of treatment outcomes will be essential to improve future data quality and increase potential for data sharing, meta-analyses, and facilitating multicenter tuberculosis research to improve outcomes.

In 2018, 10 million cases of tuberculosis were reported globally [1]; tuberculous meningitis accounts for 1%–5% of these cases [2]. Tuberculous meningitis is the most severe form of tuberculosis and is responsible for a considerable burden of neurological sequelae and mortality; a systematic review of treatment outcomes in 1636 children with tuberculous meningitis estimated a mortality rate of 19.3% [3]. There is considerable variation in the reported outcomes for adult tuberculous meningitis across available studies; the reasons for this remain unclear. Two recent systematic reviews of adult tuberculous meningitis outcomes reported substantial heterogeneity in mortality and the proportion of deaths among those diagnosed with TBM, with pooled estimates of 22.8% and 24.7% [4, 5]. However, neither review attempted to explain the variation in treatment outcomes by stratifying studies by HIV status and geographical location. In addition, Wen and colleagues excluded all investigational treatment studies, effectively excluding major treatment randomized controlled trials (RCTs) investigating regimens that have now become the standard of care (eg, adjunctive steroids and delayed antiretroviral therapy [ART] for those with HIV-associated tuberculous meningitis). Furthermore, there is a paucity of data in recent meta-analyses on drug resistance rates, treatment regimens, and steroid use. HIV co-infection has been shown to be a risk factor for death (hazard ratio, 2.5; 95% CI, 1.9–3.4) in Vietnamese adults with tuberculous meningitis [6], but this remains to be explored systematically in other regions [7–9]. Neurological disability in adult tuberculous meningitis survivors has not been studied in detail in meta-analyses. In 2 recent systematic reviews, the prevalence of disability in adult tuberculous meningitis survivors varied between 29% and 50% [4, 5]. However, neither review provided data on the nature and severity of neurological sequelae in tuberculous meningitis survivors.

We performed a systematic review and meta-analysis to characterize treatment outcomes, namely all-cause mortality and neurological sequelae, for adult tuberculous meningitis across a range of epidemiological settings. We endeavored to perform a definitive review by including the best quality data available and performing a robust quality assessment of the studies included.

## METHODS

### Literature Search Strategy

This review followed the Preferred Reporting Items for Systematic Reviews and Meta-Analyses (PRISMA) statement for the reporting of systematic reviews and meta-analyses [10]. A systematic electronic search was conducted using MEDLINE and EMBASE with the aim of identifying all studies reporting treatment outcomes in adult tuberculous meningitis from 1988 to the present. This time period corresponds to the WHO recommendation of standard quadruple therapy for the treatment of tuberculosis [1]. Controlled and natural language terms identified key search concepts such as “tuberculosis,” “meningitis,” “mortality,” “complications,” and “outcome.” The full search strategies are presented in Supplementary Appendix A. Searches were conducted on July 9, 2018.

### Study Selection

A 2-stage sifting process was employed: (1) at title and abstract and (2) at full-text level according to eligibility criteria as detailed below. Sifting was performed in duplicate independently by 2 reviewers, and any unresolved disagreements were resolved by a third independent reviewer. Reference and citation checking were conducted for included articles.

Studies were eligible for inclusion if they (i) included adults (aged ≥15 years) with confirmed or suspected TB meningitis; (ii) utilized diagnostic criteria to systematically evaluate patients for tuberculous meningitis; (iii) reported on at least 1 of the following outcome measures: neurological sequelae, in-hospital mortality, mortality at the end of follow-up; (iv) employed any of the following study designs: consecutive case series, case–control study, cohort study, randomized controlled study, systematic review, or meta-analysis.

The following exclusion criteria were applied: (i) studies with <10 participants; (ii) studies limited to specific complications or comorbidities (eg, hydrocephalus, tuberculoma, or surgical intervention); (iii) studies not providing at least a backbone of standard fixed-dose combination antituberculous therapy; (iv) studies not specifying treatment given; (v) studies published before 1988; (vi) studies not written in English; (vii) any systematic review superseded by an updated systematic review; (viii) narrative reviews not adding new data or new analysis of data to existing knowledge.

### Data Extraction and Data Synthesis

Two authors independently extracted data on study characteristics, recruitment populations, and treatment outcomes from eligible studies using a standardized, piloted electronic data capture database (REDCap, Vanderbilt University, Nashville, TN, USA). We captured data on geographical region, number of HIV-positive participants, British Medical Research Council (MRC) tuberculous meningitis grade at presentation, treatment regimens utilized, use of corticosteroids, and outcomes reported at specified time points for each study. Any unresolved disagreements in extraction were resolved by a third independent reviewer.

We used each study’s definition of neurological sequelae as reported in the study. For articles that utilized the modified Rankin Scale or the Barthel index, “disability” was defined as “any disability that impeded the patient’s ability to carry out tasks they once performed.” This is was represented as a score of >2 on the Modified Rankin Scale or <80 on the Barthel Index.

For systematic reviews, individual study-level data were not extracted or analyzed; only the summary estimates were recorded for comparison, and citation checking was performed to ensure all relevant source manuscripts had been identified.

### Data Analysis

We used the proportion of all-cause deaths and neurological sequelae within each study to define outcomes of tuberculous meningitis for the meta-analyses. As such, all meta-analyses used random effects models and employed the DerSimonian and Laird method on Freeman-Tukey transformed proportions, which is the established approach for this type of analysis [11–13]. We graphically displayed data in forest plots, which display point estimates of tuberculous meningitis outcomes in each study, with 95% confidence intervals. We generated pooled effect estimates by inverse variance–weighting each individual point estimate, such that the estimates with lower variances contributed more to the pooled estimate [13]. The overall pooled estiamte for mortality was stratified by follow-up outcome reporting time. Interstudy and subgroup heterogeneity were assessed with the *I*^*2*^ statistic. All analyses were conducted in Stata, version 15.1 (StataCorp, College Station, TX, USA), with the “metaprop” command [14].

### Quality Assessment

The 39 articles included in the meta-analysis were assessed for study quality using the Downs and Black tool, a 27-item quality assessment checklist [15]. Each study was scored on a 32-point scale for items that examined quality of reporting, external validity, internal validity (bias and confounding), and study power. Study power was estimated according to sample size methodology. Studies were scored as follows; 0 if no sample size calculation was made or reported in the manuscript (given for observational studies); 3 if a power calculation was done but there were insufficient numbers of patients recruited; 5 if the power calculation was done and sufficiently powered. Systematic reviews meeting the inclusion criteria were not assessed for risk of bias. As treatment outcomes were of interest in these analyses and not treatment or intervention efficacy, we included all studies regardless of quality assessment score.

### Role of Funding Source

The Fogarty International Center of the National Institutes of Health provided funding fellowship support to the lead author of the study. The funders had no role in the study design, data collection, data analysis, data interpretation, or writing of the report. The corresponding author had full access to all the data in the study and had final responsibility for the decision to submit for publication.

## RESULTS

### Search Results, Studies, and Participants Included

**Figure 1.**
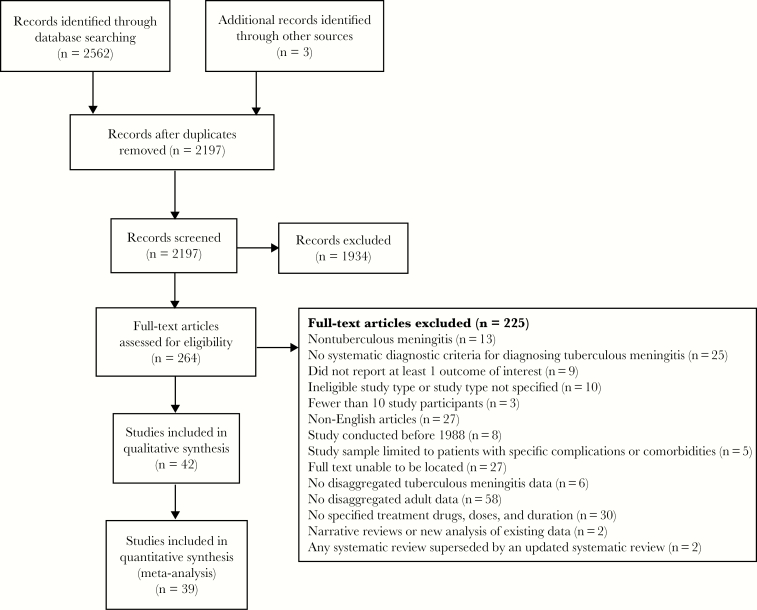
Flow diagram of the study selection process.

**Figure 2.**
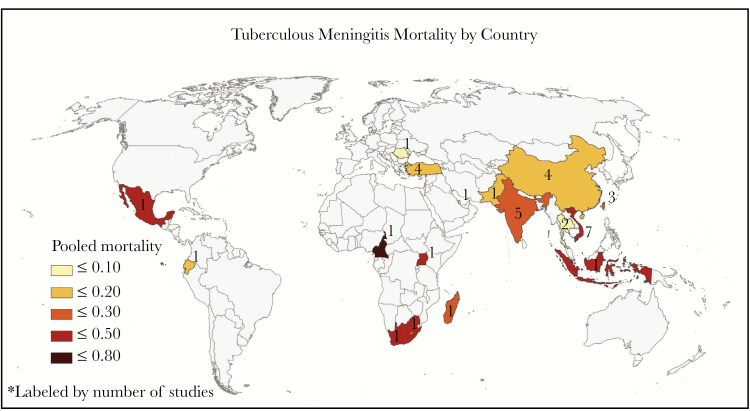
Tuberculous meningitis mortality by country. Pooled mortality for tuberculous meningitis by country. Mortality for countries with only 1 study reflect the reported mortality for that 1 study.

Our searches yielded 2562 reports. After removal of duplicates (n = 365), 2197 studies underwent title and abstract screening, and 264 full texts were reviewed (Figure 1). Thirty-nine studies met our eligibility criteria for inclusion and analysis (Table 1). These 39 studies were published between 1995 and 2018; 10 (26%) were case series, 21 (54%) were cohort studies, and 8 (21%) were randomized controlled trials. Studies arose from 18 countries, including a range of epidemiological settings; 24 (62%) were from high–TB burden settings, and 15 (38%) were from low–TB burden settings. A total of 26 (67%) studies were conducted in Asia, and 5 (13%), 5 (13%), and 2 (5%) were conducted in Europe, Africa, and the Americas, respectively (Figure 2). Study quality scores ranged from 8 to 32, with a score of 32 indicating the highest quality. The median quality score for included articles (interquartile range [IQR]) was 18 (15–20). Our meta-analysis includes reported treatment outcomes for 5752 adults with tuberculous meningitis. Participant age ranged from 15 to 88 years. Seven studies included 1078 HIV-positive patients: 302 (28%) from Africa and 776 (72%) from Asia. MRC tuberculous meningitis grade was reported in 29 studies, in which 28% (1354/4761) of participants presented with MRC grade I disease, 48% (2302/4761) with grade II, and 20% (967/4761) with grade III. A total of 37 studies (n = 5623 participants) reported the classification or uniform case definition of enrolled participants. Of those, 40% (2243/5623) were microbiologically confirmed tuberculous meningitis and 49% (2741/5623) were suspected tuberculous meningitis, the latter of which included 21% (1013/5623) with probable tuberculous meningitis and 12% (663/5623) with possible tuberculous meningitis according to the uniform case definition [16]. Only 12 studies reported on drug resistance rates; 10 studies included patients with multidrug-resistant tuberculosis (n = 49), and 12 studies included patients with mono-drug-resistant tuberculosis, of which 10 studies included isoniazid resistance (n = 112 participants), 6 studies included rifampin resistance (n = 12 participants), and 1 study included streptomycin resistance (n = 1 participant).

**Table 1.** Characteristics of Studies Meeting Inclusion Criteria

First Author	Year	Study Design	Country	No.	Diagnostic Criteria^a^	HIV, No. (%)	Confirmed TBM, No. (%)	Suspected TBM, No. (%)^b^	MDR TB, No. (%)	INH Mono-R, No. (%)	Antituberculous Treatment^c^	Steroids^c^	Outcome(s) and Time Point Reported, mo
Africa													
Luma [17]	2013	Case series	Cameroon	54	2 a b	54 (100)	1 (2)	53 (98)			2RHZE/6-8RH	All received steroids: unspecified drug(s), dose, & duration	In-hospital mortality
Marais [18]	2011	Cohort	South Africa	120	2 a b c d e f	106 (88)	47 (39)	73 (61)	3 (3)	3 (3)	RHZE	Not specified	In-hospital and 6-mo mortality
Thinyane [19]	2015	Case series	Lesotho	22	2 a b e f	15 (68)	0 (0)	22 (100)			RHZE	Not given	Mortality at the end of follow-up
Cresswell [20]	2018	Cohort	Uganda	195	2 a b c d	106 (54)	74 (38)	93 (48)	0 (0)	0 (0)	RHZE	Not specified	In-hospital mortality
Raberahona [21]	2017	Case series	Madagascar	75	1	3 (4)	8 (11)	44 (59) probable, 23 (31) possible			2RHZE/6RH + S if prior TB (n = 2)	Not given	Mortality at 8 mo
South america													
Gonzalez-Duarte [22]	2011	Cohort	Mexico	64	2 a c f	14 (22)	44 (69)	20 (31)			2RHZE/RH – mean time of therapy was 11.9 ± 7 mo	57 (78%) received steroids, unspecified drug(s), dose, & duration	Mortality and neurological outcomes at 5 mo
Alarcon [23]	2013	Cohort	Ecuador	310	2 a b d e f g h	2 (1)	140 (45)	170 (55)			2RHZ + E or S or quinolone/10RH (quinolone given to some)	Steroids given to patients with severe disease, unspecified drug(s), dose, & duration	Mortality and neurological outcomes at 12 mo
Asia													
Torok [24]	2008	Cohort	Vietnam	58	2 b d e f	58 (100)	54 (93)	4 (7)	4 (7)		3RHZE + S if prior TB/6RH	D (0.3–0.4 mg/kg) tapered over 6–8 wk	Mortality at 9 mo
Torok [25]	2011	RCT	Vietnam	253	2 a b d e f	253 (100)	158 (62)	95 (38)	4 (2)		3RHZE + S if prior TB/6RH	D (0.3–0.4 mg/kg) tapered over 6–8 wk	Mortality and neurological outcomes at 9 and 12 mo
Heemskerk [6]	2016	RCT	Vietnam	817	1	349 (43)	407 (50)	214 (26) 174 (21)	15 (2)	86 (11)	2RHZE/6RH + S if prior TB + L in 1 trial arm	D (0.3–0.4 mg/kg) for 6–8 wk	Mortality at 9 mo
Thwaites [26]	2002	Cohort	Vietnam	56	2 a b d	11 (20)	56 (100)	0 (0)	0 (0)	3 (5)	3RHZE/6RHZ if HIV+ 3RHZS/6RHZ if HIV-	Not given	Mortality at 3 mo
Thwaites [27]	2004	RCT	Vietnam	545	2 a b d e f	98 (18)	187 (34)	358 (66)	10 (2)		3RHZS/6RHZ 3RHZE/6RHZ if HIV+ or prior history of TB	D (0.3–0.4 mg/kg) tapered over 4 wk, then oral treatment (4 mg/d) tapered for 4 wk	Mortality and neurological outcomes at 9 mo
van Laarhoven [8]^d^	2017	Cohort	Indonesia	608	2 a b c d	93 (15)	336 (55)	272 (45)			RHZE (n = 47: high-dose R; n = 25: M instead of E)	91% received steroids; drug, dose, and duration not specified	Mortality at 12 mo
Singh [28]	2016	Cohort	India	141	1	13 (9)	54 (38)	87 (62)			2RHZS/7HE	D (0.3–0.4 mg/kg) tapered over 4 wk, then oral treatment (4 mg/d) tapered for 4 wk	Neurological outcomes at 9 mo
Tai [29]	2016	Cohort	Malaysia	36	1	3 (8)	23 (64)	13 (36)			2RHZE/10RH	Not specified	Neurological outcomes at 3 mo
Chen [30]	2014	Cohort	Taiwan	38	2 b d f g	2 (5)	Not reported	Not reported			2RHZE/10-16RHE	D (12–16 mg), P (60–80 mg) tapered over 6–8 wk	Mortality and neurological outcomes at 18 mo
Kalita [31]^e^	2014	RCT	India	60	2 a b c d e f	3 (5)	24 (40)	36 (60)			RHZE	P (0.5 mg/kg/d) for 1 mo, tapered over 4 wk	Mortality and neurological outcomes at 6 mo
Sheu [32]	2012	Case series	Taiwan	91	2 b c d e f g	3 (3)	Not specified	Not specified			RHZE +/- S	Either D 12–16 mg/d or P 60–80 mg/d over 1.5–2 mo	In-hospital mortality and neurological outcomes
Wasay [33]	2014	Case series	Pakistan	404	2 a b d e f g h	1 (0.2)	35 (9)	369 (91)	2 (0.5)		RHZE + 8% (n = 34) received S	Unspecified regimen given to all	Mortality and neurological outcomes at 2 mo
Chotmongkol [34]	1996	RCT	Thailand	59	2 a	0 (0)	6 (10)	53 (90)			2RHZS/4RH	29 (52%) P 60 mg tapered over 5 wk	Mortality and neurological outcomes at 6 and 18 mo
Lu [35]	2001	Cohort	China	36	2 a c d e f	0 (0)	23 (64)	13 (36)			RHZE +/- C and/or S for drug toxicity	Unspecified steroid given to patients with clinical deterioration	Mortality and neurological outcomes at 3 and 6 mo
Wang [36]	2002	Cohort	China	41	2 a d f g	0 (0)	22 (54)	19 (46)	0 (0)	0 (0)	RHZE	Unspecified steroid given to 9 patients	Mortality at 6 mo
Chotmongkol [37]^f^	2003	Cohort	Thailand	45	2 a b d	0 (0)	2 (4)	42 (93)			2RHZS/4RH	Not given	Mortality at 6 mo
Thwaites [38]	2003	Cohort	Vietnam	21	2 a b d e f g	0 (0)	15 (71)	6 (29)	0 (0)		3RHZS/6RHZ	Not given	Mortality and neurological outcomes at 9 mo
Malhotra [39]	2009	RCT	India	91	2 a b e f	0 (0)	18 (20)	73 (80)			2RHZE or S/7RH	D (0.3–0.4 mg/kg) tapered over 4 wk, then oral treatment (4 mg/d) tapered for 4 wk OR MP 5 d OD of either 1 g (weight >50 kg) or 20 mg/kg (<50 kg)	Mortality and neurological outcomes at 6 and 18 mo
Hsu [40]	2010	Case series	Taiwan	108	2 a d c e f g h	0 (0)	46 (43)	62 (57)	1 (1)	2 (2)	6RHZ + S, C, or L in case of toxicity or side effects	P (minimum 20 mg) for >7 d given for 1 to >4 wk in n = 106	Mortality at 9 mo
Sharma [41]	2013	Case series	India	42	2 a e f g	0 (0)	4 (10)	38 (90)			RHZE	6 wk of steroids, unspecified drug(s) & dose	Mortality and neurological outcomes at 6 mo
Sun [42]	2014	Cohort	China	33	2 a d e f h	0 (0)	7 (21)	26 (79)			RHZE +/- PAS + L if in trial arm 2	D 1.5–15 mg/d for 1.5–6 wk	In-hospital neurological outcomes
Kalita [43]	2014	Case series	India	34	2 a b c d e f h	0 (0)	34 (34)	0 (0)			9RHZE/9RH	P (0.8 mg/kg, max 40 mg) for 1 mo	Mortality and neurological outcomes at 6 mo
Imam [44]	2015	Case series	Qatar	80	2 a b c d e f g h	0 (0)	35 (44)	45 (56)			RHZE + 4% received S, M, and A	D (med 21 mg/d) P (med 40 mg/d) over 3–9 wk	Mortality and neurological outcomes at 12 mo
Zhang [45]	2016	Cohort	China	401	1	0 (0)	131 (33)	202 (50)	4 (1)	6 (1)	RHZE + L	Not specified	5-y mortality
Kalita [46]	2016	RCT	India	57	2 a b d e f h	0 (0)	18 (32)	39 (68			6RHZE + L in trial arm/12RH for following year	P (0.5 mg/kg/d) for 1 mo tapered over 1 mo	Mortality and neurological outcomes at 3 and 6 mo
Li [47]	2017	Case series	China	154	1	0 (0)	18 (12)	98 (61) probable, 42 (27) possible			2-4RHZE/6-12RH	D (early treatment), unspecified dose & duration	Mortality and neurological outcomes at 8 mo
Mai [48]	2018	RCT	Vietnam	120	1	0 (0)	92 (77)	26 (22)	1 (0.1)	10 (8)	3RHZES/6RH	D (0.3–0.4 mg/kg) tapered over 4 wk, then oral treatment (4 mg/d) tapered for 4 wk	Mortality and neurological outcomes at 2 and 8 mo
Europe													
Cagatay [49]	2004	Cohort	Turkey	42	2 a b d e f g h	2 (5)	10 (24)	32 (76)			3-6RHZE	D (8 mg) for 4–6 wk given to patients who were stage II or III	Mortality at 12 mo
Doganay [50]	1995	Cohort	Turkey	72	2 a b d f	0 (0)		72 (100)			51%: 2RHZS/6RH 49%: various combinations 12–16 mo R, H, Z, E, S	P or D 4–6 wk if MRC stage 3 diseases/signs of raised ICP	Mortality at 2 y
Sutlas [51]	2003	Cohort	Turkey	61	2 b d e f g h	0 (0)	19 (31)	42 (69)			1RHZES/2-3RHZE/4-9RHZ (if no tuberculoma present)/10-12RH	P (1 mg/kg/d) for 1 mo, tapered for 4 mo	Mortality at 12 mo
Sengoz [52]	2008	Cohort	Turkey	121	2 a b d e f g h	0 (0)	52 (43)	69 (57)	4 (3)		2RHZ + E or S/7-10RH	2D (16 mg/d) for those with neurological deficits	Mortality at the end of follow-up
Miftode [53]	2015	Cohort	Romania	127	1	0 (0)	25 (20)	35 (28) probable, 70 (55) possible			2-3RHZE/7-9RH	All received: unspecified drug, dose, & duration	In-hospital mortality and neurological outcomes

Abbreviations: TB treatment: Number of months placed in front of regimen code: A, amikacin; C, ciprofloxacin; D, dexamethasone; E, ethambutol; H, isoniazid; L, levofloxacin; M, moxifloxacin; P, Prednisolone; R, rifampicin; S, streptomycin; Z, pyrazinamide.

^a^Diagnostic criteria legend: 1= uniform case definition, 2 = other criteria used to diagnose and categorise patients, including a = suggestive CSF picture, b = microscopy, c = Xpert/PCR, d = culture, f = evidence of extraneural TB, g = response to treatment, h = other (history of TB or contact with a TB-infected individual, positive mantoux reaction, IGM AB in the CSF, biopsy, etc.) ^b^Some participants were considered “suspected” as well as “confirmed” TBM. ^c^TB treatment (given to all unless specified otherwise): number of months placed in front of regimen code: R = rifampicin, H = isoniazid, Z = pyrazinamide, E = ethambutol, S = streptomycon, L = levofloxacin, M = moxifloxacin, C = ciprofloxacin, A = amikacin, PAS = paraaminosalacylic acid, P = prednisolone, D = dexamethasone, MP = methylprenisolone. Where no duration of antituberculous therapy or steroids is stated, it means it was not clearly specified in the paper. ^d^van Laarhoven et al. includes some data from 3 clinical trials in Indonesia [54–56]. The primary studies were excluded from the review to avoid duplication of data.

^e^Only included participants who were treated with RHZE. ^f^Treatment information was taken from Chotmongkol [34], as they were from the same authors, hospital, and decade.

The most common treatment regimen was standard 4-drug therapy of rifampicin, isoniazid, pyrazinamide, and ethambutol (RHZE) with no additional antituberculous drugs (n = 17 studies). Seven studies used streptomycin in addition to or in replacement for ethambutol (Table 1). The median treatment duration (IQR) was 9 (9–12) months. Corticosteroids were given to all patients in 19 studies and to some participants in 10 studies (Table 1). Treatment outcomes by corticosteroid use were examined in a meta-analysis with included studies, but this was not the aim or design of our meta-analysis, and a significant amount of heterogeneity in mortality between studies was unexplained (Supplementary Appendix B). A Cochrane meta-analysis on corticosteroid use in TBM was published in 2016 [57].

### Mortality Assessment and Outcomes

A wide range of mortality end points were reported: 15% (6/39) studies reported 1-month mortality, 5% (2/39) studies reported 2-month mortality, 8% (3/39) studies reported 3-month mortality, 18% (7/39) studies reported 6-month mortality, 13% (5/39) studies reported 12-month mortality, and 2% (1/39) reported 5-year mortality. Other reported outcomes included in-hospital mortality (n = 6 studies) and median time to death (n = 4 studies). In the 6 studies that reported on “in-hospital mortality,” only 1 study reported on the length of hospitalization, which ranged from 4 to 10 days until death or discharge. Five studies did not define “in-hospital mortality” in terms of time frame.

**Figure 3.**
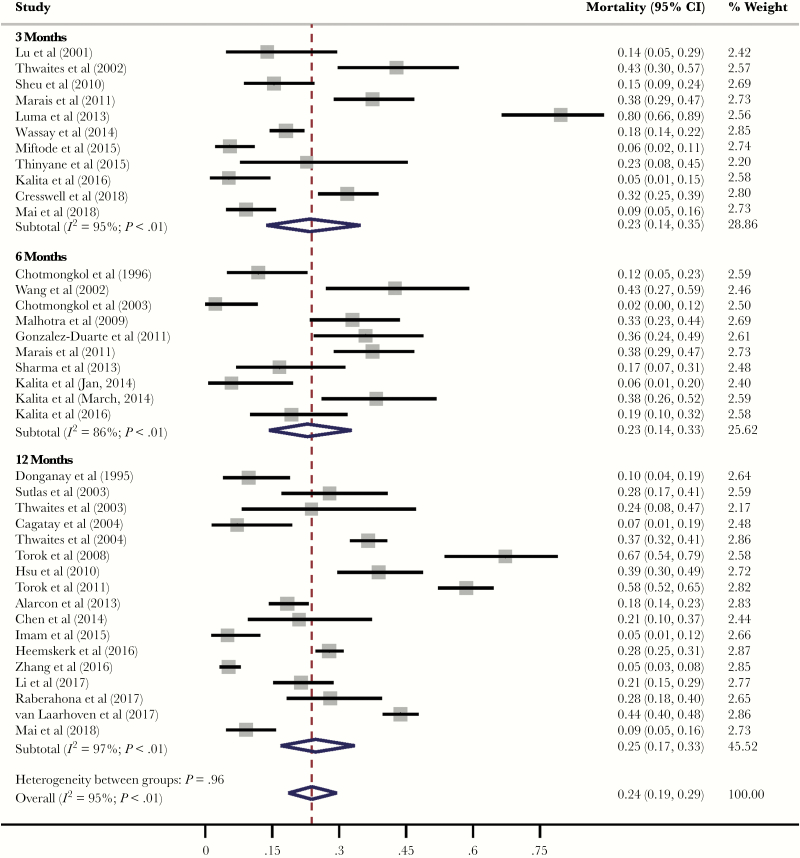
Tuberculous meningitis mortality by outcome reporting time point. Forest plots depicting mortality due to tuberculous meningitis at 3, 6, and 12 months. One study was excluded because the outcome time point was not reported.

To investigate time-specific mortality, articles were grouped by follow-up outcome reporting time point. Articles that reported outcomes ≤3 months were included in the 3-month reporting category to summarize “early” mortality. Articles that reported outcomes >3 months to 6 months were included in the 6-month reporting category. Articles that reported outcomes >6 months were included in the 12-month reporting category. Of articles reporting outcomes at 3, 6, and 12 months, pooled mortality was 23% (95% CI, 14%–35%), 23% (95% CI, 14%–33%), and 25% (95% CI, 17%–33%), respectively (Figure 3). There was significant heterogeneity (*I*^2^ = 95%; *P* < .01) for all outcome reporting time points. There was no marked heterogeneity in mortality between outcome reporting time points (*P* = .60), but it was included in the pooled analysis, resulting in a pooled mortality of 24% (95% CI, 19%–29%).

### Mortality End Points by HIV Status

**Figure 4.**
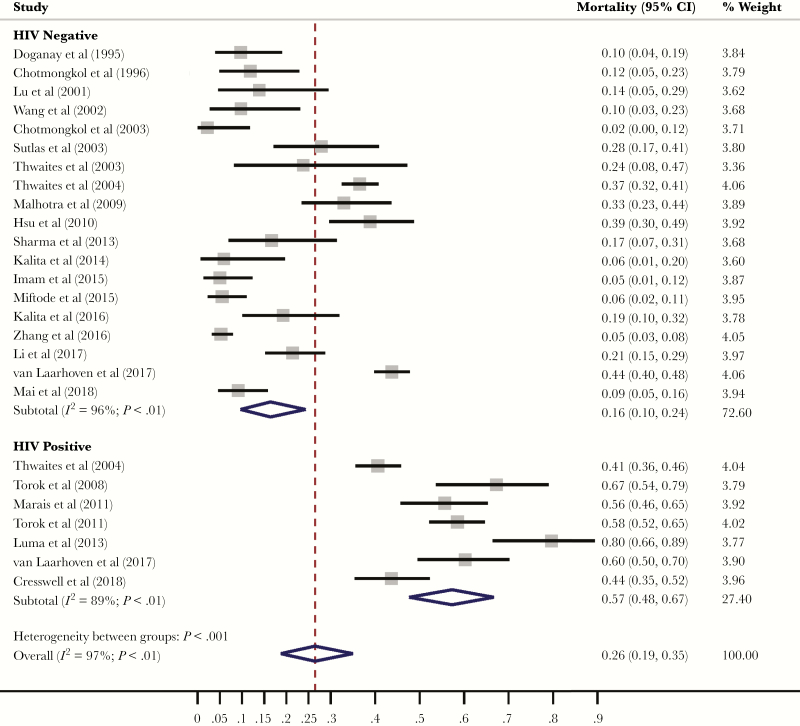
Tuberculous meningitis mortality by HIV status. Forest plot depicting mortality due to tuberculous meningitis stratified by HIV status. HIV status explains a significant amount of the heterogeneity in tuberculous meningitis mortality (*P* < .01).

Seven studies reported mortality for HIV-positive adults. For HIV-positive adults, pooled mortality was 57% (95% CI, 48%–67%), compared with 16% (95% CI, 10%–24%) in HIV-negative adults (Figure 4). HIV status explained a significant amount of the observed heterogeneity in tuberculous meningitis mortality (*P* < .01).

### Mortality End Points by Geographical Region

Most studies reporting on tuberculous meningitis mortality were conducted in India and the Asian continent (n = 27; 70%), where pooled mortality ranged from 2% to 67% (Figure 2). The countries reporting the highest tuberculous meningitis mortality were located in Sub-Saharan Africa, where mortality ranged from 23% to 80%. Continent (Africa vs Asia) explained a significant amount of the observed heterogeneity in tuberculous meningitis mortality (*P* = .02).

### Temporal Variation in Mortality End Points

To investigate changes in tuberculous meningitis treatment outcomes over time, we conducted a temporal analysis in which individual studies were allocated to 1 of 5 time periods and stratified analyses were conducted. Time periods were subdivided into 5-year windows from 1995 onwards, and pooled mortality was analyzed within each time window. The highest pooled mortality was 31% (95% CI, 14%–51%) in articles published from 2006 to 2010, though there was no significant variation by time window (Supplementary Appendix C). In earlier time periods, heterogeneity in survival was greatest, and heterogeneity appears to have declined in more recent time periods.

### Neurological Disability

Functional outcomes among survivors was a prespecified end point in 24 studies; 10 studies reported on functional outcomes using the Modified Rankin Scale score (n = 6) or the Barthel Index (n = 5), 10 studies reported on neurocognitive disability without using a specified scale or measurement tool, and 5 studies reported using “clinical assessments.”

The timing and method of neurological assessments varied between studies; the most commonly used outcome assessment was physical disability conducted at the end of follow-up. In this analysis, participants were considered disabled if there was any indication of functional disability as reported by the Modified Rankin Scale or Barthel Index. Of the studies utilizing the Modified Rankin Scale, the pooled proportion of patients experiencing some level of physical disability was 26% (95% CI, 18%–35%), with considerable heterogeneity (Supplementary Appendix D). Of the studies using the Barthel Index, the proportion of patients experiencing some level of physical disability was 32% (95% CI, 22%–43%), with only moderate heterogeneity.

## DISCUSSION

In this rigorous systemic review and meta-analysis, we reviewed treatment outcomes for >6000 adults with tuberculous meningitis, and our data clearly demonstrate that the mortality and neurological sequelae associated with tuberculous meningitis remain unacceptably high. Although there was significant heterogeneity between studies (*I*^2^ > 95%), the overall risk of death was 23% at 3 months and 25% at 12 months. In patients who did survive, neurological sequelae were common, affecting nearly one-third of all patients. Furthermore, our temporal analysis of treatment outcomes indicates that prognosis has improved little over time. Our results are in concordance with 2 recently published systematic reviews, which reported overall morality associated with adult tuberculous meningitis to be 23% and 25% and risk of neurological sequelae to be 29% and 50%, respectively. Our study expands on the current literature through subgroup meta-analyses to evaluate differential treatment outcomes by HIV status and geographical region.

We have demonstrated that patients with HIV-associated tuberculous meningitis have 3-fold higher mortality compared with HIV-negative cohorts; mortality in HIV-negative cohorts ranged between 10% and 24%, compared with 48%–67% in HIV-positive cohorts (*P* < .01). Pathogenesis research is urgently needed to investigate the disproportionate mortality associated with HIV co-infection in tuberculous meningitis and to identify potential interventions or preventative measures.

Second, our data demonstrate that despite adoption of standardized treatment regimens for tuberculous meningitis, considerable global disparities in treatment outcomes exist. Pathogenesis work has shown that even within a Vietnamese population, a single genetic polymorphism significantly impacts corticosteroid responsiveness and survival from TBM [58]. The extent of the heterogeneity observed in this meta-analysis raises the possibility that genetic or other latent factors may contribute to outcome, and the current one-size-fits all approach to treatment may be effective in some individuals/populations and less effective in others. Our subgroup meta-analyses indicate that patients in the African continent have a higher mortality compared with all other continents. This may in part be explained by the higher co-prevalence of HIV. However, given the considerable resource limitations including a lack of intensive care facilities typical of many settings in Sub-Saharan Africa, it is likely that the management of commonly encountered complications of tuberculous meningitis including hyponatremia, raised intracranial pressure, hydrocephalus, stroke, and nosocomial infections is suboptimal. Further research is needed to determine the attributable mortality due to a lack of supportive or critical care in Sub-Saharan Africa. Our systematic literature review highlights the historical paucity of clinical studies published from this continent. In order to address the devastatingly poor outcomes from HIV-associated meningitis, particularly for those in Sub-Saharan Africa, we need to design, fund, and deliver more clinical research.

Our meta-analyses of follow-up time-specific mortality at 3, 6, and 12 months highlight that >90% of tuberculous meningitis deaths occur in the first 3 months. This may justify that 3-month mortality is a reasonable RCT end point, potentially making study trial follow-up shorter and cheaper, thereby accelerating research outputs. However, the considerable heterogeneity found in these analyses, as well as inconsistencies in reporting outcomes, indicates that further evidence is needed to justify a 3-month clinical trial end point. Clinical studies to identify drivers of early mortality in tuberculous meningitis may inform the design of treatment intensification strategies and other adjunctive interventions.

Concerningly, our results demonstrate that minimal improvements in survival have been made over time. There are a number of temporal factors that may have affected outcomes in certain time periods including the height of the HIV epidemic in the 1990–2005 period: ART rollout in the 1995–2010 window, the increasing availability of more rapid diagnostics in the form of the Xpert MTB/Rif assay in the 2010–2020 window facilitating the diagnosis of tuberculous meningitis where it was previously unconfirmed, and, lastly, gradually increasing rates of antituberculous drug resistance worldwide. Reporting bias, which may have varied over time, must also be considered.

Our analysis has several limitations. First, although we only included studies that employed a prespecified diagnostic criterion for tuberculous meningitis, there was considerable variation in the quality of diagnostic criteria used, and diagnostics have changed over time. We chose not to restrict diagnostic criteria to microbiologically confirmed tuberculous meningitis because doing so would have restricted our meta-analysis to 40% (n = 2243) of adults, and furthermore we wanted our results to be generalizable to real-world clinical settings where confirmation rates are often only moderate. We do, however, recognize that misclassification of undifferentiated meningitis cases as tuberculous meningitis is common, especially when left to physician discretion, as may have been the case in some of the patients included in our meta-analysis, which would undermine the accuracy of our outcome estimates. Second, in the spirit of generalizability, we chose to include case series, which are primarily descriptive and not wholly representative of the populations they are drawn from. Although this may have posed some unmeasurable bias, we believe that this would not have substantially impacted our results, as mortality and neurological sequelae, our outcomes of interest, would not have measured differently or changed based on study design. Third, the specific antituberculous regimen utilized and drug resistance rates within the cohorts were inconsistently reported in studies; therefore, we were unable to conduct stratified meta-analyses based on drug resistance patterns. The International Tuberculous Meningitis Research Consortium paper on standardized methods for enhanced quality and comparability of tuberculous meningitis studies specifies that it is essential to document the dose, route of administration, and duration of all antituberculosis drugs used in tuberculous meningitis studies [59]. There remain several outstanding questions concerning the optimal treatment of tuberculous meningitis, and therefore to facilitate cross-study comparisons and interrogate differences in study outcomes, basic information about the treatment provided is essential.

Finally, there was a considerable lack of standardization of reporting on treatment outcomes. This was particularly marked with respect to reporting of neurological sequelae; first, neurological sequalae were rarely reported (only 10/39 [26%] studies including any data on neurological sequalae), the tools used were inconsistent (9 tools in total), and the time points for assessment were rarely reported. This inconsistent reporting hampered comparison of data across studies. Given the importance of neurological disability in tuberculous meningitis and the importance of developing a standardized evidence base against which to assess new treatments, the International Tuberculous Meningitis Research Consortium recommends that the Modified Rankin Score be used as the first-line tool, which should be recorded at 12 months from antituberculosis treatment initiation in all adults [59]. We support this recommendation, and in addition we would suggest that mortality be routinely reported on at 3, 6, and 12 months, if possible, to improve study comparability.

The strengths of this work include its size, with 39 individual studies included from Asia, Africa, Europe, and the Americas, making our estimates broadly generalizable to a range of settings. Our systematic review is larger than 2 previously published systematic reviews of adult tuberculous meningitis [4, 5]. In comparison to Wen et al. [4], we decided to include randomized controlled trials in our systematic review, which enabled us to include the highest quality of trial evidence, and we also reported drug resistance rates within each included study. In comparison to Wang et al. [5], we ascertained variation in treatment outcomes geographically and reported on the nature and severity of reported neurological sequelae. Overall, we assessed a wide range of covariates to investigate the heterogeneity in treatment outcomes observed. To our knowledge, this is the most extensive critical appraisal of tuberculous meningitis outcomes to date.

In conclusion, adult tuberculous meningitis is associated with considerable neurological morbidity and mortality and remains a major challenge in TB-endemic regions. The worst outcomes are observed by those with HIV co-infection in Sub-Saharan Africa, where risk of death is 3-fold higher. Our study was limited by suboptimal reporting on diagnostic criteria utilized, drug resistance rates, and details of treatment regimens used, as well as highly variable outcome reporting. Adoption of standardized reporting systems across tuberculous meningitis studies would not only facilitate cross-study comparisons, but would also improve the quality of research outputs overall and support collaborative research across centers with an aim of improving tuberculous meningitis outcomes globally.

## Supplementary Data

Supplementary materials are available at *Open Forum Infectious Diseases* online. Consisting of data provided by the authors to benefit the reader, the posted materials are not copyedited and are the sole responsibility of the authors, so questions or comments should be addressed to the corresponding author.

ofaa257_suppl_Supplementary_appendix
