# Supplementary material for: Treatment Outcomes in Adult Tuberculous Meningitis: A Systematic Review and Meta-analysis
Source: Open Forum Infect Dis. 2020 Jun 30;7(8):ofaa257. doi: 10.1093/ofid/ofaa257 (PMC7423296; doi:10.1093/ofid/ofaa257)
Supplement: ofaa257_suppl_Supplementary_appendix [file ofaa257_suppl_supplementary_appendix.docx]

**Appendix A: Search Strategy**

| **#** | **Database** | **Search term** | **Results** |
| --- | --- | --- | --- |
| 1 | Medline | "TUBERCULOSIS, MENINGEAL"/ | 6850 |
| 2 | Medline | ((TB OR tubercul*) ADJ2 mening*).ti,ab | 6694 |
| 3 | Medline | ((TB OR tubercul*) ADJ2 (brain OR cerebral OR neurological)).ti,ab | 929 |
| 4 | Medline | (1 OR 2 OR 3) | 9169 |
| 5 | Medline | exp MORTALITY/ | 343596 |
| 6 | Medline | exp "TREATMENT OUTCOME"/ | 876952 |
| 7 | Medline | exp "OUTCOME AND PROCESS ASSESSMENT (HEALTH CARE)"/ | 968126 |
| 8 | Medline | exp "SURVIVAL ANALYSIS"/ | 257144 |
| 9 | Medline | "TUBERCULOSIS, MENINGEAL"/mo | 192 |
| 10 | Medline | "TUBERCULOSIS, MENINGEAL"/co | 1175 |
| 11 | Medline | (sequalae OR sequelae OR complication* OR outcome* OR death* OR mortality OR survival).ti,ab | 3381437 |
| 12 | Medline | (5 OR 6 OR 7 OR 8 OR 9 OR 10 OR 11) | 3965816 |
| 13 | Medline | (4 AND 12) | 2762 |
| 14 | Medline | exp ADULT/ | 6572439 |
| 15 | Medline | (adult* OR age OR aged).ti,ab | 3017488 |
| 16 | Medline | (14 OR 15) | 7975296 |
| 17 | Medline | (13 AND 16) | 1593 |
| 18 | EMBASE | "TUBERCULOUS MENINGITIS"/ | 6409 |
| 19 | EMBASE | ((TB OR tubercul*) ADJ2 mening*).ti,ab | 5995 |
| 20 | EMBASE | ((TB OR tubercul*) ADJ2 (brain OR cerebral OR neurological)).ti,ab | 831 |
| 21 | EMBASE | (18 OR 19 OR 20) | 8847 |
| 22 | EMBASE | exp *MORTALITY/ | 129762 |
| 23 | EMBASE | exp *"TREATMENT OUTCOME"/ | 69616 |
| 24 | EMBASE | exp *SURVIVAL/ | 89772 |
| 25 | EMBASE | *FATALITY/ | 4759 |
| 26 | EMBASE | exp *COMPLICATION/ | 237190 |
| 27 | EMBASE | *"ADVERSE OUTCOME"/ | 4524 |
| 28 | EMBASE | (sequalae OR sequelae OR complication* OR outcome* OR death* OR mortality OR survival).ti,ab | 4751087 |
| 29 | EMBASE | (22 OR 23 OR 24 OR 25 OR 26 OR 27 OR 28) | 4928804 |
| 30 | EMBASE | (21 AND 29) | 2534 |
| 31 | EMBASE | exp *ADULT/ | 97301 |
| 32 | EMBASE | (adult* OR age OR aged).ti,ab | 4369238 |
| 33 | EMBASE | (31 OR 32) | 4393218 |
| 34 | EMBASE | (30 AND 33) | 969 |

**Appendix B**: Tuberculous Meningitis Mortality by Steroid Use

**Appendix C**. Tuberculous Meningitis Mortality by Year Published

Forest plots depicting the variation of tuberculous meningitis mortality by year published. Statistical analysis of variation of mortality over time was not possible, but there appears to be considerable variation.

**Appendix D**. Physical Disability by Modified Rankin Scale and Barthel Index

Proportion of enrolled patients experiencing some disability as indicated by the modified Rankin Scale (score >2) and Barthel Index (score <80)
